# Optimization of Deep Learning Network Parameters Using Uniform Experimental Design for Breast Cancer Histopathological Image Classification

**DOI:** 10.3390/diagnostics10090662

**Published:** 2020-09-01

**Authors:** Cheng-Jian Lin, Shiou-Yun Jeng

**Affiliations:** 1 Department of Computer Science and Information Engineering, National Chin-Yi University of Technology, Taichung 411, Taiwan; shiouyun@ncut.edu.tw; 2 School of Intelligence, National Taichung University of Science and Technology, Taichung 404, Taiwan

**Keywords:** breast cancer, histopathology, deep learning, convolutional neural network, uniform experimental design

## Abstract

Breast cancer, a common cancer type, is a major health concern in women. Recently, researchers used convolutional neural networks (CNNs) for medical image analysis and demonstrated classification performance for breast cancer diagnosis from within histopathological image datasets. However, the parameter settings of a CNN model are complicated, and using Breast Cancer Histopathological Database data for the classification is time-consuming. To overcome these problems, this study used a uniform experimental design (UED) and optimized the CNN parameters of breast cancer histopathological image classification. In UED, regression analysis was used to optimize the parameters. The experimental results indicated that the proposed method with UED parameter optimization provided 84.41% classification accuracy rate. In conclusion, the proposed method can improve the classification accuracy effectively, with results superior to those of other similar methods.

## 1. Introduction

Breast cancer is a commonly diagnosed cancer in women worldwide. In Taiwan (with a population of 23 million), 1 in 120 women are diagnosed as having breast cancer annually, and the breast cancer incidence is increasing [1]. The accuracy of histopathological image classification is essential for early breast cancer diagnosis. The techniques of breast cancer diagnosis depend on investigation of histopathological images such as mammography, magnetic resonance imaging (MRI), ultrasound, positron emission tomography (PET), thermography, and surgical incision [2,3]. However, in the early stages, detecting breast cancer from histopathological images is difficult because these images cannot convey warning signs and symptoms [4]. Therefore, various computer-assisted systems have been developed to overcome the drawbacks of histopathological image analysis. Generally adopted workflows in computer-aided diagnosis image tools for breast cancer diagnosis have focused on quantitative image analysis [5]. Recently, advanced engineering techniques have been used by research groups such as the Visual Geometry Group and Google, which have modeled the VGG-16, ResNet and GoogleNet models [6]. These engineering techniques include deep learning models based on convolution neural networks (CNNs), used to improve breast cancer diagnosis efficiency [7]. For instance, the public dataset Breast Cancer Histopathological Database (BreakHis) comprises microscopic images under different magnifying factors of breast tumor tissues collected from patients, with each sample labeled as either benign or malignant [8]. However, Lin et al. [9] achieved an accuracy rate of 83% by using BreakHis. This was achieved by optimizing the hyperparameters. This study uses deep learning networks based on a CNN with parameter optimization to improve the accuracy achieved in studies using BreakHis for image classification.

In medicine, deep learning networks achieve outstanding results in image analysis applications. CNN, a deep learning network type, has emerged as a powerful tool in the automated classification of human cancer histopathology images [10]. The LeNet-5 system represents an effective network for CNN application, with a high recognition rate. Other deep learning networks include single-layer CNN [11], RF classifier + PFTAS [12], LeNet-5(Sgdm) [13], LeNet-5(Adam) [14], and LeNet-5(RMSprop) [15]. The training of a CNN requires excessive computations with large sample and parameter settings to solve practical problems [16]. This enables for a reduction in the number of network computing samples and meets the basic parameter settings for CNN application. The most applicable CNN model is identified through experimentation [17,18]. In addition, Lin et al. [19] used a uniform experimental design (UED) to determine network parameters and improved the overall accuracy. Zhou et al. [20] used a UED to obtain parameters optimization of the formula of Xiaokeyinshui extract combination treating diabetes and to assess predicted values of selected equations in optimized doses of herb extracts. However, this study uses a uniform experimental design (UED) and optimizes the CNN parameters of breast cancer histopathological image classification.

To optimize CNN parameters, UED—A technique based on probability theory, mathematical statistics, and statistical experimental design [21]—was used to reduce the computation time of the experiment. UED can be used to select representative sample sets and arrange all possible experimental parameters found in few experiments uniformly distributed within the parameter space [22]. A series of UED tables indicate that the number of levels is equal to the number of experimental runs [23]. In addition, UED evaluates the factors affecting the results within a minimum number of experiments to obtain sufficiently accurate predictions [24]. Wang et al. [25] used UED to optimize parameters and obtain valuable results from few experiments. In this study, the UED method is used to optimize the parameters of CNN architecture for the application of breast cancer histopathological image classification.

To enhance the classification accuracy, the current study developed a CNN based on UED to solve the complicated parameter setting problem. The main purpose of this study was to use UED to optimize CNN parameters for breast cancer histopathological image classification. Therefore, the main contribution of this study used the UED method to find the optimal parameter combination of the CNN architecture for performing the fewest required experiments and time. In the UED method, the regression analysis was used to find optimization parameters in CNN architecture. Experimental results show that the proposed CNN based UED parameter optimization surpasses the existing techniques with high accuracy, making it more practical in clinical diagnosis.

The rest of this paper is organized as follows: Section 2 introduces the deep learning networks and UED parameter optimization. Next, Section 3 presents the experimental results. Finally, Section 4 provides the current conclusions and future recommendations.

## 2. Materials and Methods

**Figure 1:**
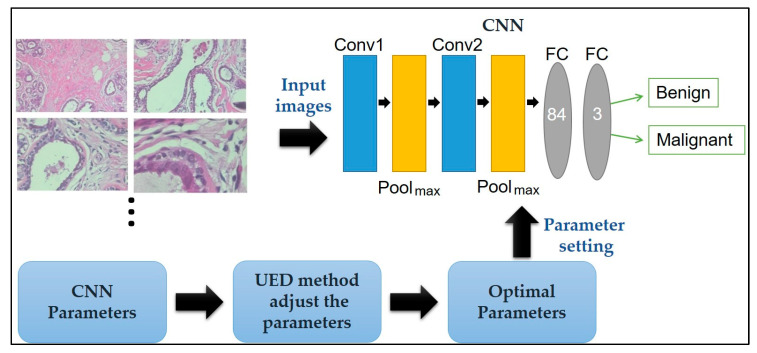
Framework of the proposed method for breast cancer histopathological image classification.

Here, a CNN paired with UED parameter optimization is proposed to improve classification performance. The framework of the proposed method is illustrated in Figure 1. Herein, the framework of the proposed method is discussed: The breast cancer histopathological images in BreakHis is presented in Section 2.1, the CNN model’s ability to classify benign and malignant tissue is described in Section 2.2, and the UED method’s ability to adjust the parameters of the CNN architecture and evaluate the optimal parameter combinations is presented in Section 2.3.

### 2.1. Materials

In this study, the experimental images were collected from BreakHis, for which the breast tissue samples were obtained from 82 patients at Pathological Anatomy and Cytopathology Laboratory in Brazil [8]. For each patient, several breast tissue samples were aspired using a fine biopsy needle in the operating room. Each sample was prepared as follows: First, formalin fixation and embedding in paraffin blocks was performed to preserve the original tissue structure and its molecular composition. Then, the 3-μm-thick sections were cut from the paraffin blocks on a high precision microtome. Finally, the sections were mounted on covered glass slides for visualization under light microscope [26]. 

**Figure 2:**
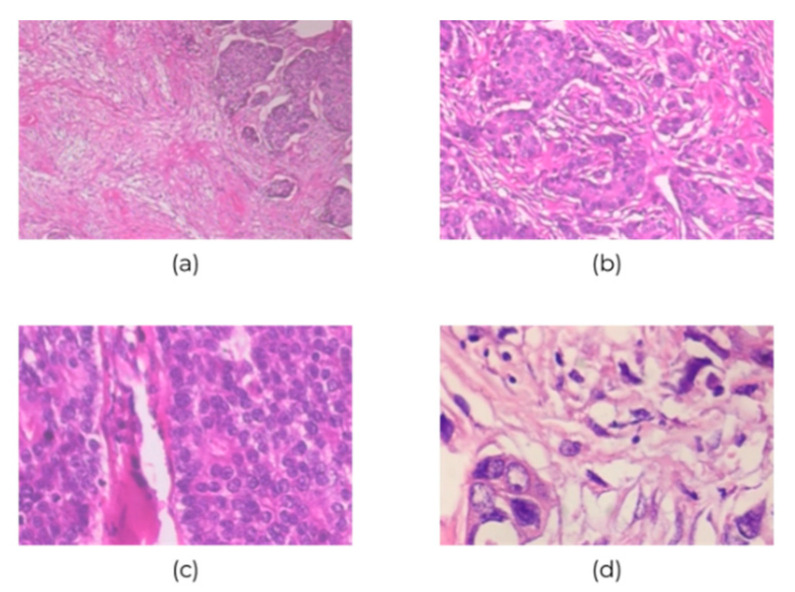
Sample images of breast cancer histopathological at (**a**) 40×, (**b**) 100×, (**c**) 200×, and (**d**) 400× magnification.

BreakHis contains 7909 700×460-pixel histopathological images of breast cancer at four ascending magnifications (40×, 100×, 200×, and 400×); of them, 2480 and 5429 images are of benign and malignant cancers, respectively. Table 1 and Figure 2 show the 3-channel RGB images with 8-bit color depth in each channel and the various magnifications. However, the class imbalance issue could bias the discriminative capability of CNN classification; this is the BreakHis dataset limitation, and it would tend towards predicting images as malignant. Therefore, the collected data were divided into training and validation sets: The first 70% of images were for training the network, and the remaining 30% were for validating it.

### 2.2. The CNN Architecture

**Figure 3:**
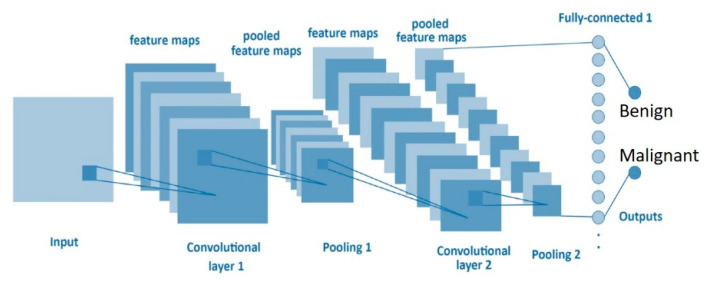
Convolutional neural network (CNN) structure.

A CNN based on deep learning networks learns a hierarchy of increasingly complex features by successive convolution, pooling, and nonlinear activation operations [27,28]. This study designed the architecture of a CNN based on the LeNet network structure including an input three-layer convolutional layer, a two-layer max-pooling layer, a fully connected layer, and final classification. The kernel size, stride, padding, and filters are described in Figure 3 and Table 2.

(a) Input layer: The input image size in this study was 50 × 50 × 3, and the first convolutional layer has six filters of 5 × 5-sized feature maps from the previous layer to input layer.
(b) Convolutional layer: Here, the network architecture contained three convolutional layers. The second convolutional layer has 16 filters of 5 × 5-sized feature maps connected to the previous layer. The third convolutional layer has 120 filters of 1 × 1-sized feature maps used from the previous layer. In our model, the stride is 1, so the size of zero padding (*zp*) is given by the following formula:
  (1) $$zp=\frac{k-1}{2}$$
  where *k* is the filter size.
(c) Pooling layer: According to the network architecture of LeNet, two pooling layers were inserted between the three convolutional layers. This experiment did not adjust the parameter of the pooling layer and maintains its size of 2 × 2.
(d) Output layer: A fully connected layer adopts the ReLU nonlinear function, which is used to categorize samples as benign or malignant. To compute the output size for a convolutional layer, we adopt the following formula:
  (2) $$os=\frac{w-k+2p}{s}+1$$
  where *w* is the input size. The padding is *p* = 0 as the padding and convolution are performed in two separate steps. The ReLU activation function is defined by the following formula:
  (3) f(x)=max(0,x)
  where *x* is the inputs of a neural network.

### 2.3. UED Method

**Figure 4:**
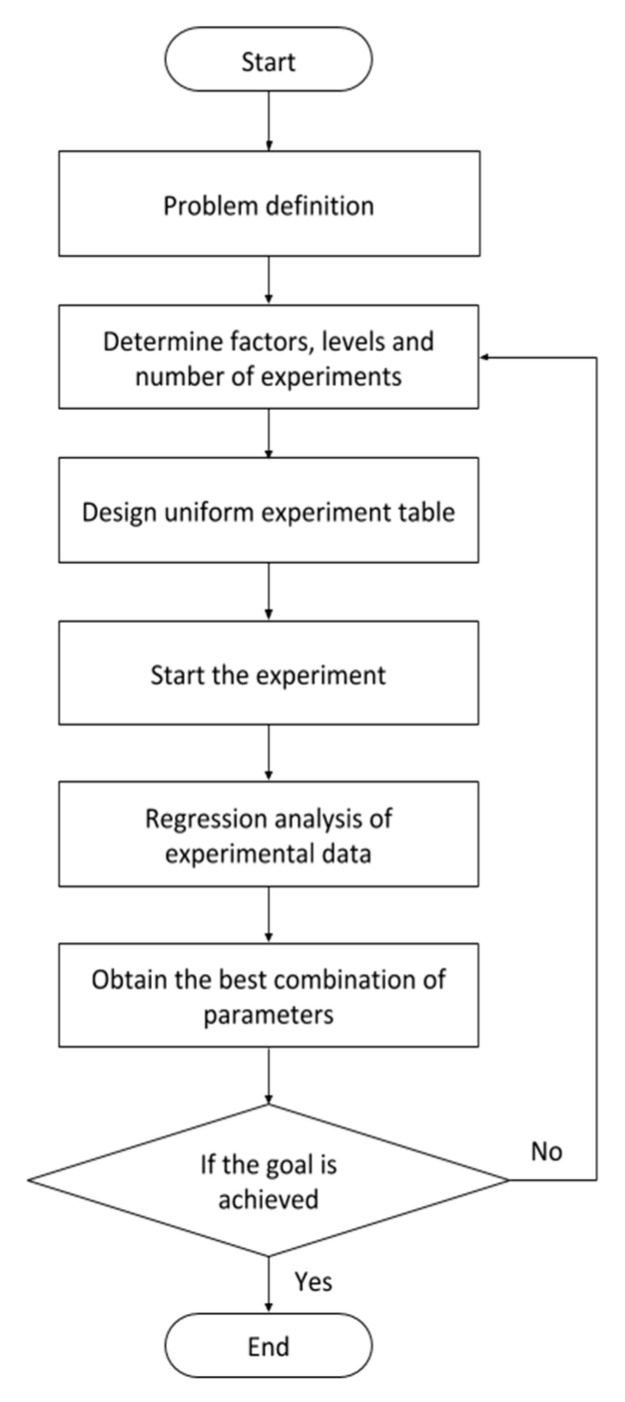
Uniform experimental design (UED) method flow.

In this study, the UED method is proposed to adjust the parameters of the CNN architecture and find the optimal parameter combinations. The UED method is used to replace the combination of all possible experimental parameters with few experimental trials uniformly distributed within the parameter space [29]. The flow of the UED method is illustrated in Figure 4. 

Step 1: Define the experimental conditions.

Step 2: Determine the factors, levels, and numbers of experiments.

Eight affecting factors and mixed levels 2 and 3 are identified herein: A–D and E–H represent first and second convolution layers parameters, respectively. The factors, levels and number of experiments are provided in Table 3.

Step 3: Design a uniform experiment table.

High-level designs and the corresponding optimization methods used to construct the designs have smaller CD^2^ values. This assumption allows for the measuring of a design, the uniformity of the double design in terms of the centered L2-discrepancy (CD^2^) and wrap-around L2-discrepancy (WD^2^), and sets the lower bounds of the centered L2-discrepancy in double designs [30,31]. UED tables are used to evaluate the data from UED experiments. The form of UED tables is defined by $U_{n}(q^{s})$, where *U* denotes uniform design; *n* the number of runs, *s* the number of factors, and *q* the number of levels [32]. A *U*_17_ (17^8^) design table was used to arrange the experiments, where *U* represents the uniform design, the subscripted 17 the test number, the superscripted 8 the maximum factor number, and 17 the level number. Table 4 and Table 5 present CD^2^, WD^2^, and *U*_17_ (17^8^) values, with the least deviation.

Step 4: Start the experiment.

Step 5: Analyze the experimental data.

To find the optimization parameters for using a regression analysis during the optimization process, the response variables are fitted by a quadratic model [17], as shown in Equation (1).

(4) $$\varepsilon =Y-[\;\alpha_{0}+\;\sum_{i=1}^{n}\alpha_{1i}X_{i}+\;\sum_{i=1}^{n}\alpha_{2i}X_{i}^{2}+\;\sum_{i=1}^{n}\alpha_{3i}X_{i}^{3}+\;\sum_{i=1}^{n-1}\sum_{j=i+1}^{n}\alpha_{4ij}X_{i}X_{j}\;]$$

where ε is the error, *Y* is the accuracy, $X_{i}$ the factors, *n* the number of affecting factors, $\alpha_{0}$ the constant, and $\alpha_{1i},\alpha_{2i},\alpha_{3i},\;\mathrm{and}\;\alpha_{4ij}$ the coefficients of *X*.

Step 6: Obtain the best combination of parameters.

Step 7: End the experiment if the goal is achieved; otherwise, repeat the experiment beginning from step 2.

## 3. Experimental Results

In this experiment, the UED method was used to optimize the CNN architecture. The minimum number of experiments was 17 to evaluate the parameter optimization of the CNN. BreakHis is used to verify breast cancer histopathological image classification. The uniform layout (UL) of U_17_ (17^8^) was used to allocate the eight factors with 17 levels as shown in Table 6. Table 7 provides the observed results for each experiment. The three tests could be performed with the identical parameter combination, and each observation was recorded independently. The average classification accuracy of the CNN is 83.4% on run 2 of this experiment. In addition, by using UED based on regression analysis for parameter optimization combination of CNN architecture, the average classification accuracy of the optimized structure in BreakHis has improved by 1.01% (Table 8). Therefore, the optimal parameter combination for the conv1_Kernel size, conv1_Filter, conv1_Stride, conv1_Padding, conv2_Kernel size, conv2_Filter, conv2_Stride, and conv2_Padding is 7, 12, 2, 1, 3, 8, 1, and 1, respectively.

**Figure 5:**
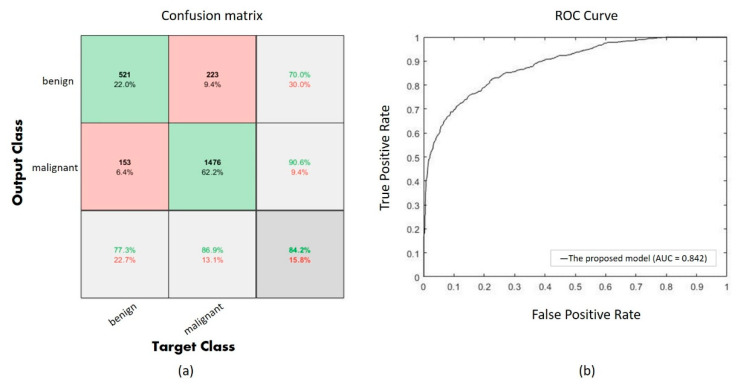
(**a**) The confusion of the proposed model, (**b**) the ROC curve of the proposed model.

Performance of the proposed CNN with UED parameter optimization is evaluated using a confusion matrix as shown in Figure 5a, and the ROC curve is also shown in Figure 5b. The confusion matrix shows 1997 correct classifications (521 of benign and 1476 of malignant) among 2373 validation images, and the AUC is 0.842 in the ROC curve.

Table 9 provides a comparison of the proposed CNN paired with UED parameter optimization and the alternative methods, such as the single-layer CNN [9], RF classifier + PFTAS [10], LeNet-5(Sgdm) [11], LeNet-5(Adam) [12], LeNet-5(RMSprop) [13], and CNN with Taguchi method [7]. This table illustrates that the accuracy of the optimized network architecture is 84.41%, and it has an accuracy superior to other methods.

Comparison results of advanced engineering techniques, such as the VGG-16, ResNet-101 and GoogleNet, are shown in Table 10. The highest average accuracy rate of GoogleNet is 85.46%, but the computational time for training is 33 min 27 s. However, the proposed CNN with UED parameter optimization obtains 84.41% average accuracy rate, and the computational time for training is only 13 min 41 s and fewer than other methods.

## 4. Conclusions

This study proposes UED parameter optimization for deep learning networks used to perform experiments on breast cancer histopathological image classification. In the proposed UED approach, uniform experiment table and regression analysis were used to adjust CNN architecture to optimize the parameter combination. Based on the experimental design in this study, the optimal parameter combination of a CNN is achieved when the optimum parameters are as follows: the conv1_Kernel size, conv1_Filter, conv1_Stride, conv1_Padding, conv2_Kernel size, conv2_Filter, conv2_Stride, and conv2_Padding is 7, 12, 2, 1, 3, 8, 1, and 1, respectively. The experimental results indicated that the average accuracy of the proposed method (using BreakHis) is 84.41%, and this is 1.01% higher than the accuracy of a CNN without using UED. In addition, the classification accuracies of the proposed method were 6.91%, 3.13%, 3.72%, 2.19%, 1.83%, and 1.22% higher than the single-layer CNN, RF classifier + PFTAS, LeNet-5(Sgdm), LeNet-5(Adam), LeNet-5(RMSprop), and CNN with Taguchi methods, respectively. The experimental results present that the proposed CNN based on UED parameter optimization improves the network performance and is superior to other methods.

The contributions of this study include providing users in modeling with a small number of experiments to find the most efficient parameter combination of CNN architecture, thus reducing experimental times and improving classification accuracy. The limitations of this study are that only the first and second convolutional layers are used as affecting factors. Nevertheless, a CNN with UED parameter optimization demonstrated future learning potential and can process the variable size of training and test data sets. Future studies should focus on the optimal size of input patches for deep learning algorithm development of new architectural structures. This enables researchers to efficiently identify the factors influencing parameter optimization and potentially consider multiple-input CNNs in the future. In addition, the limitation of the BreakHis dataset is the imbalance issue between benign and malignant dataset. Therefore, we will used Generative Adversarial Network (GAN) model to extend the benign dataset in the future work.

## Figures and Tables

**Table 1:** BreakHis dataset image distribution in terms of class and magnification factor.

Magnification	Benign	Malignant	Total
40×	652	1370	1995
100×	644	1437	2081
200×	623	1390	2013
400×	588	1232	1820
Total of Images	2480	5429	7909

**Table 2:** The proposed CNN architecture.

Layer	Image Size	Kernel Size	Stride	Padding	Filter
Input	50 × 50 × 3				
Convolution Layer 1		5 × 5	1	0	6
Relu Layer					
MaxPooling Layer1		2 × 2	2 × 2		
Convolution Layer 2		5 × 5	1	0	16
Relu Layer					
MaxPooling Layer2		2 × 2	2 × 2		
Convolution Layer 3		1 × 1	1	0	120
FullyConnectedLayer					84
FullyConnectedLayer					3

**Table 3:** Affecting factor parameters of the CNN.

No.	Factors	Level 1	Level 2	Level 3
A	conv1_Kernel size	3	5	7
B	conv1_Filter	4	6	12
C	conv1_Stride	1	2	
D	conv1_Padding	0	1	
E	conv2_Kernel size	3	5	7
F	conv2_Filter	8	16	32
G	conv2_Stride	1	2	
H	conv2_Padding	0	1	

**Table 4:** Squared values of centered L2-discreapncy (CD^2^) and wrap-around L2-discrepancy (WD^2^) of the U_17_ (17^8^).

Runs	CD^2^	WD^2^
17	0.061559	0.334477

**Table 5:** UED table of *U*_17_ (17^8^).

	Factors							
runs	1	2	3	4	5	6	7	8
1	1	5	6	8	8	9	17	17
2	2	12	17	5	15	5	10	8
3	3	10	4	15	4	6	6	4
4	4	2	9	12	14	16	3	11
5	5	16	12	2	5	12	14	13
6	6	6	10	1	10	2	4	3
7	7	13	1	17	11	14	12	9
8	8	4	16	9	1	15	11	5
9	9	15	5	10	17	3	7	15
10	10	7	2	4	3	10	1	12
11	11	8	11	14	16	11	16	2
12	12	1	13	16	6	4	9	14
13	13	14	7	6	7	17	8	1
14	14	17	15	13	9	8	2	7
15	15	3	3	3	13	7	13	6
16	16	11	8	11	2	1	15	10
17	17	9	14	7	12	13	5	16

**Table 6:** UL of U_17_ (17^8^) used to allocate the eight factors with 17 levels.

	Conv_1				Conv_2			
Exp. No	A	B	C	D	E	F	G	H
1	5	12	1	0	3	32	1	0
2	5	12	1	1	3	32	2	1
3	3	4	1	0	7	8	2	1
4	3	12	1	0	3	16	2	0
5	7	6	2	0	7	8	1	0
6	3	12	1	1	5	16	1	1
7	7	6	1	0	7	32	2	0
8	7	4	2	1	7	32	1	0
9	3	6	2	0	5	16	2	0
10	5	4	1	1	7	32	2	1
11	5	4	1	1	5	8	2	1
12	5	6	1	0	3	16	1	1
13	7	4	2	0	5	8	1	0
14	5	12	2	1	5	8	2	1
15	3	4	2	1	3	8	2	0
16	7	6	2	1	3	8	1	0
17	3	6	2	0	5	16	1	0

**Table 7:** Results of the CNN in BreakHis.

	Factor										Result	
*Run*	*1*	*2*	*3*	*4*	*5*	*6*	*7*	*8*	*Y*_1_ (%)	*Y*_2_ (%)	*Y*_3_ (%)	*Y_avg_* (%)
*1*	5	12	1	0	3	32	1	0	82.81	82.81	81.12	82.24
*2*	5	12	1	1	3	32	2	1	82.55	83.99	83.65	83.40
*3*	3	4	1	0	7	8	2	1	77.20	82.55	82.47	80.74
*4*	3	12	1	0	3	16	2	0	83.27	82.89	82.72	82.96
*5*	7	6	2	0	7	8	1	0	72.73	82.39	77.83	77.65
*6*	3	12	1	1	5	16	1	1	82.93	74.55	82.72	80.07
*7*	7	6	1	0	7	32	2	0	83.27	81.84	83.19	82.76
*8*	7	4	2	1	7	32	1	0	82.30	80.83	83.48	82.20
*9*	3	6	2	0	5	16	2	0	82.01	82.17	83.86	82.68
*10*	5	4	1	1	7	32	2	1	82.34	83.06	83.27	82.89
*11*	5	4	1	1	5	8	2	1	82.34	81.04	75.94	79.77
*12*	5	6	1	0	3	16	1	1	83.35	82.09	75.43	80.29
*13*	7	4	2	0	5	8	1	0	77.71	82.39	77.45	79.18
*14*	5	12	2	1	5	8	2	1	79.39	83.02	83.10	81.84
*15*	3	4	2	1	3	8	2	0	82.13	82.89	83.40	82.81
*16*	7	6	2	1	3	8	1	0	82.60	83.65	81.88	82.71
*17*	3	6	2	0	5	16	1	0	75.05	81.88	81.96	79.63

**Table 8:** Comparison results between run 2 and the UED with the best parameters in the experiment.

Run	Parameter of Factors								Performance (%)
2	5	12	1	1	3	32	2	1	83.40
UED	7	12	2	1	3	8	1	1	84.41

**Table 9:** Comparison results of the various methods.

Method	Accuracy (%)
Single-layer CNN [9]	77.50
RF classifier + PFTAS [10]	81.28
LeNet-5(Sgdm) [11]	80.69
LeNet-5(Adam) [12]	82.22
LeNet-5(RMSprop) [13]	82.58
CNN with Taguchi method [7]	83.19
Our method	84.41

**Table 10:** Comparison results of advanced engineering techniques.

Method	Accuracy	Computational Time
VGG-16	84.28%	40 min 2 s
ResNet-101	84.11%	92 min 49 s
GoogleNet	85.46%	33 min 27 s
Our method	84.41%	13 min 41 s
